# The Story of the Insane from Year to Year

**Published:** 1896-11-14

**Authors:** 


### THE STORY OF THE INSANE FROM YEAR TO YEAR.

### Ninth Series.

### DERBY COUNTY ASYLUM.

This asylum contained 464 patients on January 1st, 1895, and by December 31st the numbers had risen to 579. There were 229 admissions, 41 readmissions, 60 recoveries, 10 relieved, and 57 deaths. The recoveries stand at 37·5 per cent. on the admissions, and the deaths at 11·3 per cent. on the average number resident. Of the deaths 18 were caused by cerebral and spinal disease, 5 by pneumonia, 11 by consumption, 1 by enteritis, and 1 by dysentery. Of the year's admissions 31 owed their insanity to various moral causes, such as domestic trouble, religion, &c.; 8 to intemperance in drink; 57 to hereditary influence; and in 74 there was no ascertained cause. There has of late years been a steady increase in the number of Derbyshire patients, but Dr. Lindsay does not think this is indicative of an actual increase of insanity other than can be accounted for by the increase in the population of the county, and by the discharge rate not necessarily keeping pace with the admission rate. Probably Dr. Lindsay is right; but the question is one which is very difficult of solution. Nursing lectures have been continued during the year, and seven of the staff have passed the examination of the Medico-Psychological Society. The visitors "thank Dr. Lindsay for his unceasing energy in the management of the asylum." The average weekly cost per head was a fraction under 10s.

### THE ENNISCORTHY ASYLUM.

Like so many of the Irish asylums this one is overcrowded. With accommodation for 331 there were, on January 1st, 408 in residence, and by the end of the year this number had risen to 414. There were 58 first admissions, 23 readmissions, 40 recoveries, 9 discharged relieved, and 22 deaths. Calculated on the admissions, the recoveries stand at 49·38 per cent., and calculated on the average number resident the deaths stand at 5·28; the former is well above the average of English asylums, and the latter well below it. Only four of the deaths were caused by cerebral diseases, and the same number by phthisis. Hereditary influence accounts for 37 of the admissions, drink for 8, and moral causes for 9. There are several things in Dr. Drapes' report which deserve a larger circle of readers than can be hoped for in an asylum report. For instance, it is a great pity that the general public cannot be got to see that "the curability of insanity is inversely proportional to the time which is allowed to elapse before the patient is sent under treatment." And it is a greater pity still that his remarks on marriage could not sink deep into the hearts of his countrymen. "The pedigree of a beast," he says, "is a matter of vital importance; that of a man or woman is naught." Dr. Drapes tells us he knows of men and women who have chosen as partners people who have been patients in asylums once or oftener, and many parallel cases are within the experience of every asylum physician. Like us, he has not much hope of legislation on this subject, for the idol of individual liberty is so much worshipped. A man is allowed by law to marry his cousin, although the families may be steeped in insanity, while the same law forbids him to marry his deceased wife's sister, although there be not a trace of hereditary disease. The annual cost per head is £24 7s. 6d.

### SUSSEX COUNTY ASYLUM.

On January 1st there were 888 patients in residence, and on December 31st the numbers had fallen to 847; but this decrease was caused by the transference of 62 West Sussex patients. There were 195 admissions and 38 readmissions, 62 recoveries, 42 discharged relieved, and 107 deaths. The recoveries are 27·9 per cent. on the admissions, and the deaths are 12·4 on the average number resident; the former being below the County Asylum average, and the latter above it. Forty-six of the deaths were caused by cerebral and spinal diseases, 17 by consumption, and 5 by bronchitis. Drink accounts for 22 of the year's admissions, hereditary influence for 49, previous attacks for 46, domestic trouble for 11, and religion for 3. In spite of the discharge of nearly all the West Sussex cases the asylum is almost full again, and it is anticipated that in another two years or so it will be quite full. To remedy this state of things it is proposed to build a hospital for acute cases, additional accommodation for pauper patients, special accommodation for idiots, and accommodation for private patients. As regards the hospital, it is evident that much additional annual expense would be incurred by it; but the advantages of such a building are by no means so clear. Certainly they have not yet been demonstrated. Furthermore, the whole scheme mentioned above would add several hundred beds to an asylum already containing 877, and it is very questionable whether such an enlargement ought to be made. From the Chairman's report we learn that the asylum contains 354 patients chargeable to Brighton, and it would surely be better to dissolve the partnership between the borough and the county, and let Brighton have an asylum of its own. Large asylums are mistakes from every point of view. The committee say that Dr. Saunders "continues to carry out the onerous and responsible duties which devolve upon him with the same care and attention he has hitherto displayed." The weekly cost per head is 9s. 3¾d.

### EAST RIDING ASYLUM, BEVERLEY.

The numbers increased during the year from 346 to 363. There were 63 admissions, 10 readmissions, 17 recoveries, 4 relieved, and 29 deaths. Calculated on the admissions, the recoveries stand at 26·98 per cent., and, calculated on the average number resident, the deaths are 8·05 per cent. Nine of the deaths were from cerebral diseases, 3 from pneumonia, 1 from intestinal ulceration, and 6 from consumption. Intemperance in drink drove 8 of the year's admissions insane, hereditary tendency accounts for 14, uterine disease for 13, and various moral causes for 8. The asylum is about to be enlarged so that it will contain 100 additional patients, and it is estimated that the cost will be £10,000. The committee say that the management of the asylum is excellent from every point of view. The weekly charge to the unions is 8s. 2d. There are a few private patients who pay from 13s. to 30s. a week.

